# Insertion of Exogenous Genes within the *ORF1a* Coding Region of Porcine Astrovirus

**DOI:** 10.3390/v13112119

**Published:** 2021-10-21

**Authors:** Yanjie Du, Teng Liu, Yifeng Qin, Qinting Dong, Ying Chen, Kang Ouyang, Zuzhang Wei, Weijian Huang

**Affiliations:** College of Animal Science and Technology, Guangxi University, No. 100 Daxue Road, Nanning 530005, China; yanjiedu0212@163.com (Y.D.); 13786531073@163.com (T.L.); jianke2365987@163.com (Y.Q.); dongqinting@gmail.com (Q.D.); yingchen@gxu.edu.cn (Y.C.); ouyangkang@gxu.edu.cn (K.O.)

**Keywords:** astrovirus, PASTV-GX1, DNA-launched infectious clones, HVR, transposons, tag, iLOV

## Abstract

A tagged or reporter astrovirus can be a valuable tool for the analysis of various aspects of the virus life cycle, and to aid in the development of genetically engineered astroviruses as vectors. Here, transposon-mediated insertion mutagenesis was used to insert a 15-nucleotide (nt) sequence into random sites of open reading frame 1a (ORF1a) based on an infectious full-length cDNA clone of porcine astrovirus (PAstV). Five sites in the predicted coiled-coil structures (CC), genome-linked protein (VPg), and hypervariable region (HVR) in ORF1a of the PAstV genome were identified that could tolerate random 15 nt insertions. Incorporation of the commonly used epitope tags, His, Flag, and HA, into four of the five insertion sites permitted the production of infectious viruses and allowed recognition by specifically tagged monoclonal antibodies. The results of immuno-fluorescent assays showed that Flag-tagged ORF1a protein overlapped partially with capsid and ORF2b proteins in the cytoplasm. Improved light-oxygen-voltage (iLOV) gene was also introduced at the insertion sites of CC, VPg, and HVR. Only one viable recombinant reporter PAstV expressing iLOV inserted in HVR was recovered. Biological analysis of the reporter virus showed that it displayed similar growth characteristics, and yet produced less infectious virus particles, when compared with the parental virus. The recombinant virus carrying the iLOV fused with the HVR of ORF1a protein maintained its stability and showed green fluorescence after 15 passages in cell cultures. The resultant fluorescently tagged virus could provide a promising tool for the rapid screening of antiviral drugs as well as allowing the visualization of PAstV infection and replication in living cells.

## 1. Introduction

Astroviruses are non-enveloped, positive-sense, single-stranded RNA viruses. The astrovirus genome is approximately 6.2 to 7.9 kb in length and contains 5′ untranslated regions (UTR), four open reading frames (ORF1a, ORF1b, ORF2, and ORF2b), a 3′ UTR, and a 3′ poly (A) tail [1,2,3]. ORF1a is situated at the 5′ end of the genome and encodes a nonstructural polyprotein, nsP1a, which can be proteolytically cleaved into serine proteases; a viral genome-linked protein (VPg); and other uncharacterized proteins [4,5]. ORF1b is expressed by a programmed ribosomal frameshifting mechanism and encodes an RNA-dependent RNA polymerase (RdRp). ORF2 is located at the 3′ end of the genome and encodes a capsid protein (Cap) translated from a sub-genomic RNA (sgRNA) [6]. The newly identified ORF2b overlaps with the 5′ end of the ORF2. The ORF2b protein may have a certain amount of permeabilization activity.

Astroviruses were firstly detected by Appleton and Higgins in the feces of children suffering from diarrhea in 1975 [7]. Astroviruses (AstVs) can infect most mammals and avian and are divided into two different genera, *Mamastrovirus* and *Avastrovirus*, depending on their source [1,8,9]. For human beings, AstVs mainly cause diarrhea and gastroenteritis, and they are second only to rotavirus as the leading cause of diarrhea in children. The recently reported novel human astroviruses are more closely related to animals than the eight classic Human astrovirus (HAstV) serotypes, which could have resulted from cross-species transmission events [10,11,12]. AstVs mainly cause diarrhea in susceptible people and animals [13,14,15,16]. In recent years, however, an increasing number of studies have shown that AstVs may cause neurological symptoms in humans [17], cattle [18,19,20,21], pigs [22], and sheep [23], as well as respiratory symptoms in pigs [24] and gout in goslings [25].

Reverse genetic operating system is an important tool to study the replication characteristics and pathogenic mechanisms of RNA viruses [26,27]. Full-length cDNA infectious clones for human, porcine, and duck AstVs have already been constructed [8,28,29]. A tagged or reporter recombinant AstV is a valuable tool for the analysis of various aspects of the virus life cycle and allowing rapid screening of antiviral drugs, as well as to aid in the development of genetically engineered PAstVs as vectors [30]. However, the small, compact, and overlapping genome of Astvs can complicate the reverse genetic manipulation of the AstV genome. Although targeted mutations at specific nucleotide positions have been engineered successfully into recombinant Astvs [2,3], there is no report of the recovery of viable AstV expressing heterogenous reporter proteins.

Random transposon-mediated mutagenesis is an excellent method that has been used to explore the identification of functional domains essential for virus replication, as well as insertion sites in the viral genome that can tolerate nucleotide sequence insertions [31,32]. In the present study, we used this powerful technique to produce a library of 15 nt insertions in the ORF1a protein of PAstVs. We identified five sites in ORF1a that could tolerate 15 nt insertions. Replacement of the 15 nt insertion in HVR of ORF1a by commonly used epitopes (such as His, Flag, and HA) tags allowed the production of infectious viruses and visualization of the ORF1a protein. The recombinant PAstV harboring the improved light-oxygen-voltage (iLOV) fluorescent protein was fused with HVR to allow visualization of viral replication by microscopy in the context of a complete viral life cycle.

## 2. Materials and Methods

### 2.1. Cells and Antibodies

PK-15 and BHK-21 cells were cultured as previously described [8]. PAstV-GX1 (GenBank accession: KF787112) is a PAstV type 1 strain [15]. Mouse polyclonal antibody (PcAb) against Cap protein was generated, as described in our previous study [2]. Mouse monoclonal antibodies (mAbs) against the His, Flag, HA tags, and rabbit PcAb against Flag epitope were purchased from Proteintech (Carlsbad, CA, USA). Goat anti-mouse IgG H&L (CoraLite488), goat anti-mouse IgG H&L (CoraLite594), and goat anti-rabbit IgG H&L (CoraLite594) secondary antibodies were from Proteintech (Carlsbad, CA, USA). A RNA-launched cDNA clone of PAstV type 1 strain, pMDF123, was constructed as previously described. pEGFP-C1 was purchased from Clontech (Mountain View, CA, USA).

### 2.2. Construction of DNA-Launched Infectious Clone of a PAstV Type 1 Strain

The splicing overlap extension polymerase chain reaction (SOE-PCR) was used to replace the T7 promoter with a CMV promoter. The primers used in this study are listed in Table S1. The CMV fragment was amplified with mutagenic primers using pEGFP-C1 as a template. The PCR fragment, NgO, was amplified with mutagenic primers using pMDF123 as a template. CMVNgO was prepared by SOE-PCR with outer primers (CMV-F/NgO-R) using the resulting fragments obtained from using CMV and NgO as templates. The fusional fragment (CMVNgO) was digested with SpeI and NgoMIV (NEB, Beverly, USA) and ligated into pMDF123 digested with the same restriction enzymes, creating a plasmid, pMCMV. A three-step fusion PCR was performed to amplify a hepatitis delta virus ribozyme (HDVr) element. Briefly, fragment F3-P1 was amplified by PCR with primers F3-F and P1, using pMDF123 as a template. Then, using the resulting fragment F3-P1 as a template, fragment F3-P2 was amplified by PCR with primers F3-F and P2. Finally, fragment F3-HDV was amplified by PCR with primers F3-F and P3. The fragment F3-HDV was cloned into a TOPO vector (Vazyme, Nanjing, China), generating a shuttle plasmid TOPO-F3-HDV. The HDV element was released from TOPO-F3-HDV by double-digestion with XhoI and SacII (NEB, Beverly, MA, USA), and afterwards ligated into pMCMV digested with the same restriction enzymes, resulting in a plasmid, pMCMV-HDV. All the resultant clones were analyzed by sequencing (BGI, Shanghai, China).

### 2.3. Transposon-Mediated 15 bp Random Insertion

In order to insert transposon insertions into a specific region of the PAstV genome, an HpaI-to-HindIII fragment containing half of the predicted C-terminal of coiled-coil structures (CC), genome-linked protein (VPg), and hypervariable region (HVR) of the ORF1a coding region was cloned into pMD19-T (Takara Biomedical Technology Co. Ltd., Beijing, China). The resulting plasmid was used as a template for a selectable library of replicon clones with random insertions of transposons bearing a chloramphenicol resistance gene using transposon-mediated mutagenesis, according to the manufacturer’s instructions (Thermo Fisher Scientific, Waltham, MA, USA). In brief, the target DNA clone containing a MuA transposase-mediated random insertion was selected using chloramphenicol (10 μg/mL) and ampicillin (100 μg/mL). The targeted fragments harboring transposon insertions were digested with *HpaI* and *HindIII* (NEB, Beverly, MA, USA) and then inserted into similarly digested pMCMV-HDV.

Subsequently, sub-libraries were digested with *Not I*. The *Not I* linearized sub-libraries were then closed by self-ligation to remove the chloramphenicol resistance cassette generated by the transposition reaction, in order to produce replicon sub-libraries harboring the 15 nt insertions. These sub-libraries were then transfected into BHK-21 cells. The supernatants harvested from the transfected cells were then passaged in PK-15 cells. Viral RNA at passage 3, P3 recombinant viruses, harboring the 15 nt insertions was extracted using an RNA extraction kit according to the manufacturer’s protocol. Total RNA was reverse transcribed using oligo (dT) as a primer using Moloney murine leukemia virus (M-MLV) reverse transcriptase (Takara, Beijing, China), followed by PCR amplification using primers 1606-HpaI-F and 2575-HindIII-R (Table S1). The amplified fragments were separated on a 2% agarose gel and purified using a gel extraction kit (Omega Bio-tek, Norcross, GA, USA). Purified products were then cloned into pMD-19T (Takara, Beijing, China), followed by sequencing to identify insertion sites.

### 2.4. Construction of Tags or Reporter Recombinant PAstV Infectious Clones

The 15 nt insertions in an HpaI-to-HindIII fragment were replaced with different epitope tags or fluorescent proteins (iLOV) using SOE-PCR, and the primers used are listed in Table S1. Briefly, fragments harboring epitope tags were amplified with ORF1a-1606-F/ORF1a-2575-R as the outer primers and CC/VPg/HVR-His/Flag/HA-F/R as the inner primers using pMDF123 as a template. Fragments containing *AflII* and *SalI* (NEB, Beverly, MA, USA) restriction enzymes were amplified with ORF1a-1606-F/ORF1a-2575-R as the outer primers and HVR-AflII-F/HVR-SalI-R as the inner primers using pMDF123 as a template. The amplified fragments harboring tags or the *AflII* and *SalI* restriction enzymes were then digested with *HpaI* and *HindIII* restriction enzymes and ligated into pMCMV-HDV digested with the same restriction enzymes, resulting in plasmids, pMCMV-CC/VPg/HVR-His/Flag/HA and pMCMV23-HVR-AflII/SalI, respectively. The iLOV gene was amplified with primers iLOV-AflII-F and iLOV-SalI-R and then digested with *AflII* and *SalI*. The digested iLOV was ligated into similarly digested corresponding regions of pMCMV-HVR-AflII-SalI, resulting in the production of plasmid, pMCMV-HVR-iLOV.

### 2.5. In Vitro Transcription and Transfection

The construct pMDF123 was linearized using SacII, followed by in vitro RNA transcription using the T7 RiboMAX™ Large Scale RNA Production System (Promega, Madison, WI, USA). Viruses were rescued from purified RNA and DNA, as reported previously [8]. Briefly, the purified RNA and DNA were transfected into BHK-21 cells using Lipofectamine 2000 (Invitrogen, Carlsbad, CA, USA), according to the manufacturer’s instructions. Following incubation for 6 h at 37 °C, the transfection mixture was discarded and replaced with minimal essential medium(MEM) (Gibco, Grand Island, NY, USA) containing 2% FBS for 41 h. After removal of the culture medium, the BHK-21 cells were maintained in MEM containing trypsin (0.5 µg/mL) for 1 h. After three cycles of freezing and thawing, the lysed cell mixture from the transfected cells was transferred to fresh PK-15 cells monolayer and incubated for 1 h. The medium was then replaced with Dulbecco’s modified Eagle’s medium (DMEM) (Gibco, Grand Island, NY, USA), containing trypsin (0.5 μg/mL). After incubation for 3 days at 37 °C, the supernatants from PK-15 cells were harvested and used for serial passages.

### 2.6. RT-PCR and Sequencing

Viral RNA was extracted from the supernatants of rescued viruses using an RNA extraction kit. The purified viral RNA was reversely transcribed by oligo (dT) (Tiangen Biotech, Beijing, China) as a primer using M-MLV reverse transcriptase (Takara, Beijing, China) for the synthesis of complementary DNA. The primers used to amplify the screened regions for identifying the insertion sites are shown in Table S1. Subsequently, the RT-PCR products were separated, purified, and sequenced.

### 2.7. Indirect Immunofluorescence Assay (IFA)

IFA was used to assess viral protein expression, as described previously. Briefly, the PK-15 cell monolayers were infected with parental and recombinant viruses. At 48 h.p.i, cells were washed twice with phosphate-buffered saline (PBS) and fixed with cold methanol for 15 min at 4 °C. After blocking with 1% bovine serum albumin (BSA) (Beijing Solarbio Science & Technology Co. Ltd., Beijing, China) at room temperature for 30 min, the cells were incubated with PcAb against *Cap* protein or mAbs against the His, Flag, and HA epitope tags as the primary antibodies. These were diluted 1:200 in PBS and incubated for 2 h at 37 °C, followed by incubations with secondary antibodies (either goat anti-mouse or goat anti-rabbit IgG H&L) diluted 1:500 in PBS for 1 h at 37 °C. The cell nuclei were counterstained with DAPI (Beijing Solarbio Science & Technology Co. Ltd., Beijing, China). After washing with PBS, imaging of the cells was carried out using an Olympus inverted fluorescence microscope.

### 2.8. Multi-Step Growth Curve

The multi-step growth curves of the recombinant viruses were drawn as previously described. Briefly, the PK-15 cell monolayers were infected with recombinant viruses at an MOI of 0.01 and incubated at 37 °C for 1 h. Subsequently, inoculants were discarded and cells were washed twice with PBS and then cultured in DMEM medium containing 0.5 μg/mL trypsin. The cell supernatants were then collected at indicated time-points (6, 12, 24, 36, 48, and 60 h). The viral titer at each time-point was determined by their TCID_50_ in PK-15 cells.

## 3. Results

### 3.1. Construction and Recovery of a DNA-Launched Infectious cDNA Clone of PAstV

**Figure 1:**
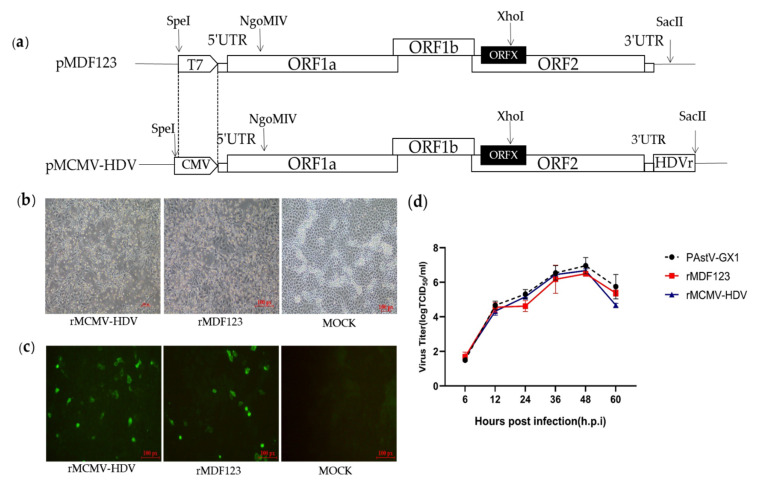
(**a**) Strategy for the construction of a full-length DNA-launched infectious clone of *PAstV.* The T7 promoter of pMD123 was replaced with a cytomegalovirus (CMV) promotor using *SpeI* and *NgoMIV*. The hepatitis delta virus ribozyme was inserted into the 3′ terminal end of the *PAstV-GX1* genome with *XhoI* and *SacII.* The restriction enzyme sites above the full-length cDNA clones are indicated by arrows. (**b**) Cytopathic effects were observed in PK-15 cells infected with rescued viruses (magnification 10×). (**c**) IFA analysis of the *Cap* protein expression in PK-15 cells. PK-15 cells infected with rescued and parental viruses were fixed at approximately 48 h.p.i and subjected to immunofluorescence using a polyclonal antibody against capsid protein. The PK-15 cells were stained with goat anti-mouse IgG H&L (CoraLite488) (magnification 20×). (**d**) Growth curves of the recombinant and parental viruses. PK-15 cells were infected with recovered and parental viruses at a multiplicity of infection of 0.01. The viral titers were determined as TCID_50_.

In a previous study, we developed an RNA-launched infectious clone of PAstV (pMDF123). In order to generate DNA-launched infectious clones of PAstV, the T7 promoter in pMDF123 was replaced with a CMV promoter and an HDVr element was inserted immediately after the viral tail region of pMDF123 in order to produce the exact 3′ end sequence of the viral genome (Figure 1a). The RNA-launched infectious clone pMDF123 and DNA-launched infectious clone (pMCMV-HDV) were then transfected into BHK-21 cells. At 48 h.p.t, the transfection products from BHK-21 cells were used to inoculate PK-15 cells for recovery of the recombinant viruses. Obvious cytopathic effects (CPEs) characteristics of rounding and detachment of cells were subsequently observed in the resultant PK-15 cells (Figure 1b).

Viral protein expression was analyzed by IFA using the a PcAb against *Cap* protein in PK-15 cells infected with rescued viruses. Expression of the viral *Cap* protein was observed in PK-15 cells infected with rescued viruses, but not in mock-infected cells. (Figure 1c). These results confirmed that the recombinant viruses could be rescued by direct transfection with infectious clones of PAstV (pMCMV-HDV). To determine the growth characteristics of the recombinant viruses, their multi-step growth kinetics were analyzed. The results showed that the recombinant viruses rescued from an infectious clone had comparable growth kinetics between 0 and 48 h.p.i to the parental viruses as well as the recombinant viruses rescued from transfected RNA (Figure 1d).

### 3.2. Identification of 15 bp Insertion Sites in the PAstV ORF1a Protein

**Figure 2:**
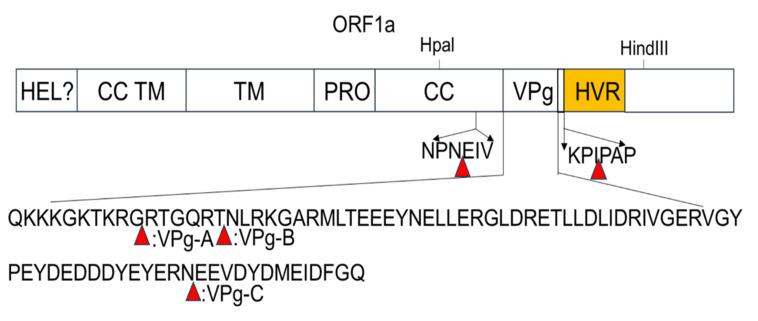
Identification of 15 bp insertion sites in the ORF1a coding region of PAstV. Schematic diagram showing the transposon-based random insertion. MuA transposase-mediated random insertion was concentrated in a specific region of the PAstV genome, an HpaI-to-HindIII fragment containing half of the C-terminal of the coiled-coil structure, as well as the predicted genome-linked protein, VPg, and the hypervariable region.

Transposon-mediated random insertion was used to identify sites in the PAstV ORF1a that would allow the insertion of a 15 nt sequence. BHK-21 cells were then transfected with the replicon sub-library harboring the 15 nt insertion in ORF1a. PK-15 cells were then infected with the transfection products at 48 h.p.t in order to rescue the recombinant viruses. Fragments encompassing the screen regions of recombinant viruses were amplified by RT-PCR and then sequenced. The results showed that five viable insertion sites were identified in the ORF1a of mutant viruses. One insertion site was located in the predicted CC structure domain; three were located in the predicted VPg region, namely, VPg-A, VPg-B, and VPg-C; and one was located upstream of the predicted HVR (Figure 2). The sites of insertion and nucleotide sequences of the 15 nt insertions are shown in Table 1.

### 3.3. Recombinant Viruses Harboring Epitope Tags Allow Visualization of the ORF1a Protein

**Figure 3:**
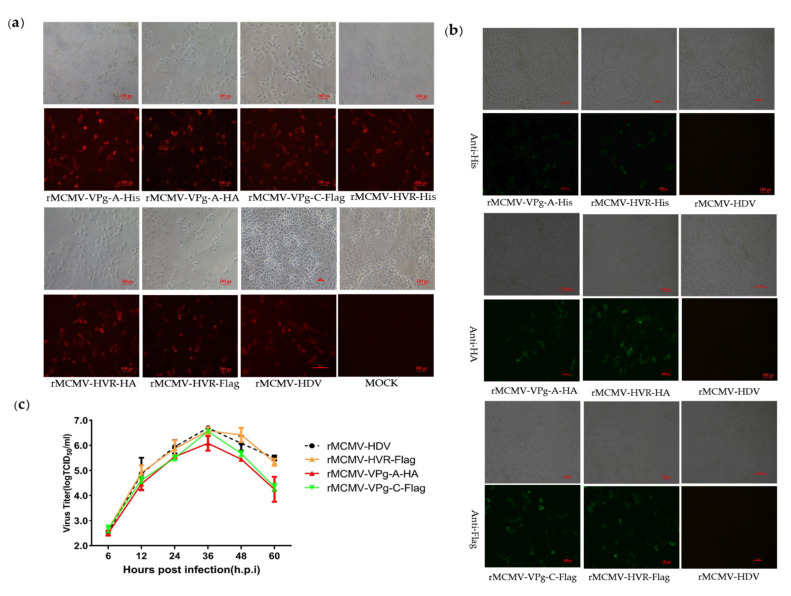
Recovery and characteristics of recombinant viruses harboring epitope tags. (**a**) IFA analysis of the *Cap* expression in PK-15 cells. Recombinant virus-infected PK-15 cells were incubated with a mouse PcAb against capsid protein and stained with goat anti-mouse IgG H&L (CoraLite594). (**b**) IFA analysis of the tags expression in PK-15 cells. The PK-15 cells were infected with recombinant virus harboring epitope tags and incubated with mouse mAbs against the His, Flag, or HA-tag. The PK-15 cell was stained with goat anti-mouse IgG H&L (CoraLite488) (magnification 20×). (**c**) Growth curves of the recombinant and parental viruses. PK-15 cells were infected with recombinant virus harboring epitope tags at a multiplicity of infection of 0.01. Cell supernatants were collected at 6, 12, 24, 36, 48, and 60 h.p.i and the virus titers were determined by their TCID_50_ in PK-15 cells.

In order to identify the viable insertion sites and visualize the expression of the ORF1a protein, the commonly used epitope tags, His, Flag, and HA, were replaced with random 15 nt sequences at insertion sites located in the predicted CC, Vpg, and HVR domains. The resulting recombinant clones harboring the epitope tags were transfected into BHK-21 cells and the supernatants obtained from the transfected cells were passaged into PK-15 cells at 48 h.p.t. A summary of rescue and tag expression of recombinant viruses harboring epitope tags is listed in Table 2. No viable viruses could be detected by RT-PCR and IFA performed in PK-15 cells inoculated with the recombinant clones harboring Flag tag in CC and VPg-A as well as different tags in VPg-B. The rescue of viruses expressing epitope-tagged proteins was first confirmed by IFA using a PcAb that recognized the Cap protein (Figure 3a). The respective expression of each epitope tag was then identified by IFA using mAbs recognizing the His, Flag, or HA tag. As shown in Figure 3b, the expression of His, Flag, or HA tags could be detected in cells infected with recombinant viruses harboring His, Flag, or HA tags fused to the HVR domain; His and HA tags in VPg-A; and the Flag tag in the VPg-C domain. No fluorescent signal was detected in PK-15 cells infected with recombinant viruses carrying tags in the CC and VPg-C domains, as well as with the parental virus.

In order to determine the replication characteristics of tag viruses, multi-step growth kinetics of the recombinant viruses were analyzed. The results showed that the tagged and parental viruses displayed comparable growth behavior between 6 to 36 h.p.i. However, the overall yield of rMCMV-VPg-A-HA was nearly 1 log less than that of the parental viruses (Figure 3c). To assess their genetic stability, the tagged viruses were passaged serially at least fifteen times. The supernatants from P3, P6, P9, P12, and P15 were collected, and the cDNA fragments containing the tag sequences were amplified by RT-PCR, followed by nucleotide sequence determination. The results showed that the tag sequences remained detectable for at least 15 cell passages (Figure S1).

### 3.4. ORF1a Protein Harboring Epitope Tags Allow Investigation of the Interactions with ORF2b Protein and Cap Protein

**Figure 4:**
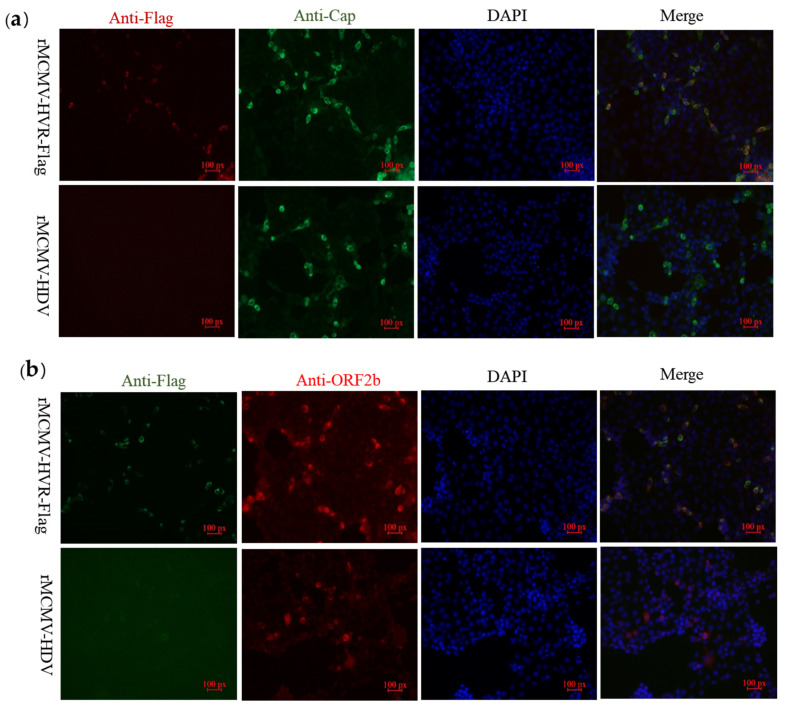
Flag-tagged ORF1a protein partially co-localizes with Cap and ORF2b proteins. (**a**) The PK-15 cells were infected with recombinant virus harboring Flag tag and parental viruses and subjected to immunofluorescence using a rabbit mAb against the Flag (red) and mouse mAb against capsid protein (green). (**b**) The PK-15 cells were subjected to immunofluorescence using a mouse mAb against the Flag (green) and a rabbit PcAb against the ORF2b protein (red). PK-15 cells were then stained with either goat anti-mouse IgG H&L (CoraLite488) or goat anti-rabbit IgG H&L (CoraLite594). The cell nuclei were counterstained with DAPI (blue) (magnification 20×).

In order to explore whether epitope tags embedded in the ORF1a protein would allow the detection of interactions between the structural proteins, ORF2b protein or Cap, and non-structural proteins coded by ORF1a, PK-15 cells were inoculated with the recombinant virus, rMCMV-HVR-Flag. IFA was performed using PcAbs against either Cap or ORF2b proteins as well as a mAb against the Flag tag. The results showed that the Flag-tagged ORF1a protein co-localized with Cap and ORF2b proteins in PK-15 cells (Figure 4).

### 3.5. Replicons Harboring iLOV in the HVR Allow Visualization of PAstV Replication

**Figure 5:**
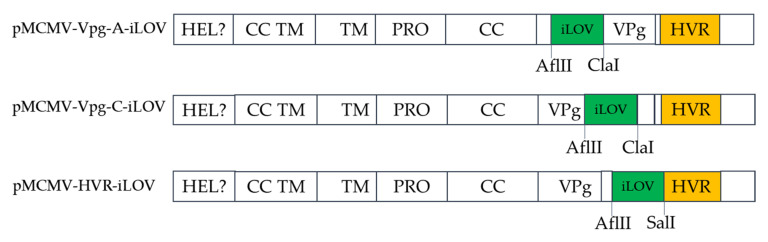
Schematic diagram showing the site of insertion of iLOV.

**Figure 6:**
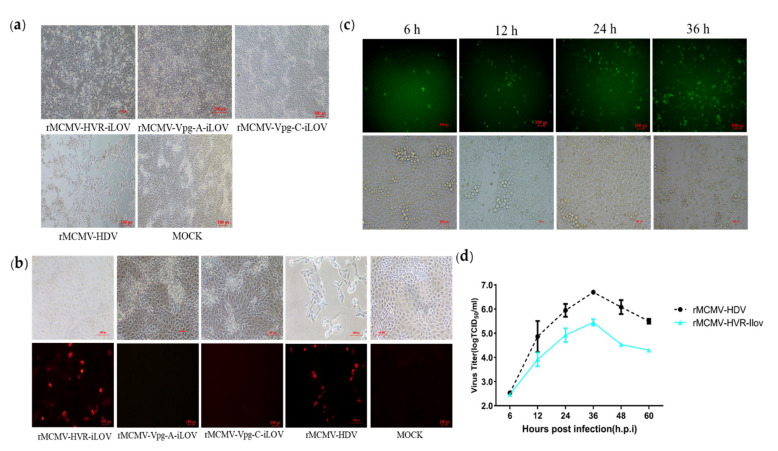
Characterization of a recombinant PAstV with insertion of the iLOV gene into the HVR. (**a**) Cytopathic effects were observed in PK-15 cells infected with recombinant reporter virus. (**b**) IFA analysis of the capsid protein expression in PK-15 cells. The PK-15 cells were infected with recombinant virus harboring iLOV and subjected to immunofluorescence using mouse PcAb against capsid protein. PK-15 cells were stained with goat anti-mouse IgG H&L (CoraLite594) (magnification 20×). (**c**) Expression of auto-fluorescent protein in cells infected with reporter virus. PK-15 cell monolayers were infected with recombinant virus harboring iLOV at a multiplicity of infection of 0.01. Green fluorescence produced from iLOV protein was directly visualized using a fluorescent microscope at 6, 12, 24, and 36 h.p.i (magnification 20×). (**d**) Growth curves of the recombinant reporter virus. PK-15 cells were infected with recombinant virus harboring iLOV at a multiplicity of infection of 0.01. Cell supernatants were collected at 6, 12, 24, 36, 48, and 60 h.p.i and the virus titers were determined by their TCID_50_ in PK-15 cells.

Efficient replication of recombinant viruses harboring epitope tags in the ORF1a protein made it possible to insert a reporter protein in order to visualize viral replication in living cells by fluorescence microscopy. To achieve this aim, a small fluorescent gene, iLOV, was inserted into the Vpg-A, Vpg-C, and HVR sites in ORF1a, as shown in Figure 5. The recombinant clones harboring the iLOV gene were transfected into BHK-21 cells. The supernatants from the transfected cells were then passaged into PK-15 cells at 48 h.p.t. CPEs were observed in PK-15 cells inoculated with supernatants from the pMCMV-HVR-iLOV-transfected cells, but not in pMCMV-Vpg-A-iLOV, pMCMV-Vpg-C-iLOV, or mock-transfected PK-15 cells, as shown in Figure 6a. In order to confirm the expression of Cap protein, cells infected with recombinant viruses were incubated with anti-Cap PcAb and then stained with a secondary antibody. As shown in Figure 6b, Cap protein could be detected in PK-15 cells inoculated with supernatants from the pMCMV-HVR-iLOV-transfected cells, but not in pMCMV-Vpg-A-iLOV, pMCMV-Vpg-C-iLOV, and mock-transfected PK-15 cells. To monitor a PAstV infection in real-time, PK-15 cells were infected with rMCMV-HVR-iLOV. The live iLOV-expressing cells at the indicated time-points were observed using a fluorescence microscope. As shown in Figure 6c, iLOV positive cells were observed in rMCMV-HVR-iLOV-infected cells as early as 6 h.p.i. There were more iLOV positive cells at 24 and 36 h.p.i, which indicated the spreading of the virus as the infection progressed. The growth kinetics were compared between the reporter and parental viruses. As shown in Figure 6d, the growth curves displayed comparable growth behavior between the reporter and parental viruses. However, the virus titer at 36 h.p.i was nearly 1 log less than that of the parental virus.

**Figure 7:**
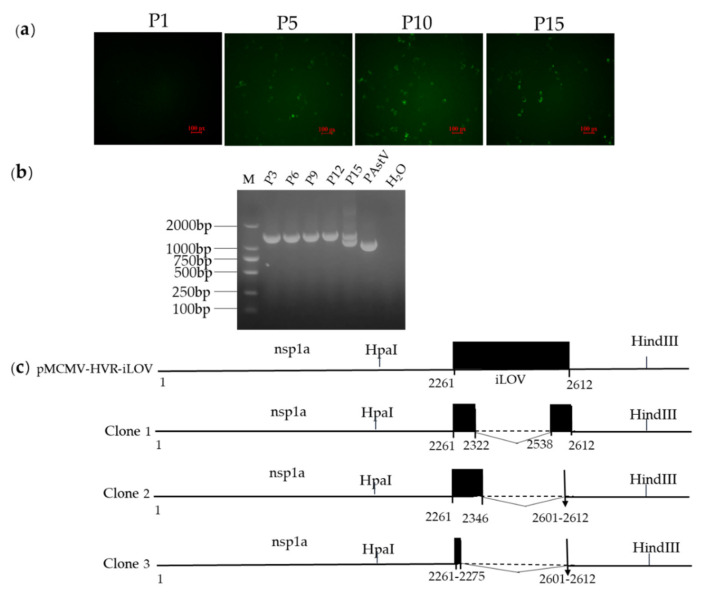
(**a**) Analysis of the stability of reporter genes in the genomes of recombinant viruses in cells. Expression of reporter auto-fluorescence during serial passages of reporter viruses. PK-15 cells were infected with P1, P5, P10, and P15 viruses. At 24 h.p.i, live cells were imaged with a fluorescent microscope. (**b**) Agarose gel pictures showing the DNA fragments covering the reporter gene as generated by RT-PCR using the RNA extracted from cells infected with P3, P6, P9, P12, and P15 viruses. (**c**) The boundaries of the nucleotide deletions detected in the reporter gene of passaged viruses. Internal deletions were found in the genomes of passaged viruses. The numbers below the boxes indicate the nucleotide boundaries of the inserts and the internal deletions. The deleted regions are indicated by the dotted lines.

In order to determine the genetic stability of the iLOV gene in the genomes of the resulting recombinant viruses, P1, P5, P10, and P15 of each reporter virus were used to infect PK-15 cells, and live cells were imaged at 24 h.p.i to evaluate the percentage of cells expressing iLOV. As shown in Figure 7a, the expression of iLOV in PK-15 cells with P5, P10, and P15 reporter virus could be directly visualized using a fluorescence microscope. The number of cells showing green fluorescence decreased markedly in P15 reporter virus-infected cells. To further identify whether the iLOV gene in the HVR sites was genetically stable during serial passages, viral RNA was extracted from P3, P6, P9, P12, and P15 viruses. Subsequently, the targeted fragments encompassing the inserted site were amplified by RT-PCR and sequenced. As shown in Figure 7b, an expected band of approximately 1 kb was detected from the parental virus RT-PCR products. An expected band of approximately 1.3 kb was obtained using P3, P6, P9, and P12 viruses as templates. A smaller PCR product of approximately 1 kb became apparent upon addition to the 1.3 kb fragment when using the P15 reporter virus RT-PCR products as templates. The sequencing results of the smaller sized PCR products showed that the P15 reporter virus produced partially deleted nucleotides in the iLOV gene (Figure 7c). These results suggested that the recombinant viruses harboring iLOV were only genetically stable for up to 12 cell passages.

## 4. Discussion

An efficient reverse genetic system can provide a reliable technical platform for studying the various aspects of the viral life cycle and pathogenesis of the virus. In a previous study, we developed an RNA-launched infectious clone of a PAstV, PAstV-GX1 strain (pMDF123) [8]. In order to improve the rescue efficiency and reduce the economic cost of RNA transcription in vitro, in the present study, we constructed a DNA-launched infectious clone of PAstV based on pMDF123. The T7 promoter of pMDF123 was replaced with a CMV promoter. Viral genomic RNA could be produced after transfection of the CMV-driven full-length cDNA plasmid into cells. An HDVr element was inserted at the end of the PAstV genome to produce the exact 3′ end sequence of the viral genome. The established CMV-driven cDNA clone was shown to infect PK-15 cells and produce infectious virions. The use of infectious clones for the rescue of AstV circumvents the need to produce viral transcripts for transfection.

The establishment of reverse genetic systems for PAstV and HAstV has created the possibility of specifically manipulating the genomes of AstVs for molecular dissection of the virus replication process. These systems have also helped in the development of genetic marker vaccines against AstVs [2,33]. However, genetic manipulation of the AstV genome is complicated by their small, compact, and overlapping genomes [1,5].

The transposon-mediated random insertion approach has been proven to be an excellent method to scan sites that can tolerate exogenous sequence insertions. This technique has been successfully applied to some positive-strand RNA viruses, such as hepatitis E virus [32], feline calicivirus [34], and hepatitis C virus [31]. The genome structure of AstV is similar to that of hepatitis E virus, in which sites within the ORF1 coding region were identified that could tolerate the insertions of tags and reporter proteins [32].

In this study, random transposon mutagenesis was applied to the ORF1a region encoding CC, VPg, and HVR in order to identify sites that could tolerate a 15 nt insertion. Five insertion sites within the ORF1a coding region were identified. One insertion site was located in the CC, three were located in the Vpg, and one was located in HVR. Subsequently, the five insertion sites were used for incorporation of different epitope tags (His, Flag, and HA) within the ORF1a protein. No viable viruses could be recovered from the recombinant clones harboring the Flag tag in CC and VPg-A as well as the other different tags in VPg-B. The amino acids’ composition and the longer sequences of the tags relative to 15 nt coding amino acids may change the secondary and higher structures of CC, VPg-A, and VPg-B. These may be involved in virus replication, which may account for the lethal effect on virus recovery, highlighting the importance of the three-dimensional structures of these predicted domains in the ORF1a protein during AstV replication.

The recombinant viruses carrying different tags in the CC, VPg, and HVR domains produced comparably less infectious viral progeny than that of parental viruses, suggesting that insertion of tags in ORF1a affect virus growth. The tagged viruses were then used for further downstream studies such as Western blotting and IFA. Unexpectedly, epitope tag expression could not be detected by IFA with mAbs against the His, Flag, or HA tags in cells infected with recombinant viruses carrying them in the CC and VPg-C domains. The epitope tags expression could not be detected by Western blotting in cells infected with any of the rescued tagged recombinant viruses (data no shown). It is possible that the respective mAbs could not recognise the tags because they were embedded in the ORF1a protein or the change in the local charge environment within this region. The proportion of cells staining for Cap or the ORF2b proteins is higher than that staining for Flag. This might be because of the nonstructural proteins coded by ORF1a, which function as replicases. These are not only expressed at low levels, but are also expressed earlier than the structural proteins after virus entry into cells. Their relatively short half-lives in cells and the relatively weak immunogenicity of ORF1a coding nonstructural proteins also made it difficult for them to be detected during infection. Functional tagging of viral proteins has been used to allow reproducible visualization of viral proteins and drug screening and evaluation [30]. In this study, we found that Flag-tagged HVR protein co-localized with Cap and ORF2b proteins in the cytoplasm, indicating that interactions between these viral proteins are closely connected.

Incorporation of different epitope tags (His, Flag, and HA) into the HVR domain allowed the production of infectious viruses as well as their recognition by the specific tags mAb. For HAstV, its HVR homology was highly distinctive, with different lengths of amino acid sequences seen in the HVR domains among the different genotypes found. Moreover, a spontaneous 45 nt deletion in the HVR was identified that was associated with the adaptation of HAstVs to cell lines, implying that this domain in the ORF1a may be an ideal site for insertion of a foreign gene [35]. The iLOV protein is an auto-fluorescent protein and has a smaller site than the widely used reporter protein GFP and RFP. Other advantages of iLOV include pH-stability and oxygen-independence. The expression of the small iLOV protein from many positive-sense single-stranded RNA viruses with small, compact, or overlapping genomes allows tracking of viral infection and replication in living cells [36,37,38]. In the present study, when iLOV was inserted into the sites identified in the CC, Vpg, and HVR domains, respectively; the only replicative and infectious recombinant clones found were the ones fused with HVR. The iLOV-positive cells were observed in reporter PAstV-infected cells as early as 6 h.p.i, with more GFP-positive cells at 36 h.p.i as the infection progressed. This indicated that the recombinant reporter PAstV allowed viral replication, which could be visualized by microscopy in the context of a complete viral life cycle.

The reporter genes in the PAstV genome were found to be genetically unstable after 15 successive passages in cultured cells. The number of cells showing green fluorescence decreased markedly in BHK-21 cell cultures infected with P15 reporter virus. The sequencing results of the smaller sized PCR products showed that the P15 reporter virus produced partially deleted nucleotides in the iLOV gene. The factors influencing the stability of the reporter gene of recombinant viruses will need further investigation. The fluorescently tagged virus could become a promising tool for the rapid screening of antiviral drugs as well as visualizing of PAstV infection and replication in living cells. Flag-tagged replicons could be used as localization immunofluorescent markers for ORF1a protein. In addition, they could be used as marker vaccines to distinguish between infected and vaccinated animals. This is the first description of the insertion of exogenous genes within the ORF1a coding region of AstV based on the transposon-mediated random insertion technique.

## 5. Conclusions

The transposon-mediated random insertion was applied to identify sites that would allow the insertion of a 15 nt sequence. Five sites in the predicted CC, VPg, and HVR in ORF1a of the PAstV genome were identified that could tolerate random 15 nt insertions. Incorporation of the commonly used epitope tags, His, Flag, and HA, into four of the five insertion sites permitted the production of infectious viruses, and allowed recognition by specifically tagged monoclonal antibodies. Introduction of iLOV at the insertion sites of HVR permitted the production of infectious viruses. The recombinant virus carrying the iLOV fused with the HVR of ORF1a protein maintained its stability and showed green fluorescence after 15 passages in cell cultures. The fluorescently tagged virus could be used as a tool for the visualization of PAstV infection and replication in living cells.

## Figures and Tables

**Table 1:** Positions of the 15 nt insertions in the ORF1a coding region.

Predicted Domain	Site of Insertion (nt)	Nucleotide Sequence of 15 nt Insertion
CC	1902/1903	CTAATtgcggccgcactaatGAGAT
VPg-A	2016/2017	GTGGGtgcggccgcagtgggCGCAC
VPg-B	2033/2034	CGGACtgcggccgcacggacCAATC
VPg-C	2199/2200	CGCAATtgcggccgcagcaatGAGGA
HVR	2261/2262	GCCCATtgcggccgcacccatCCCTG

15 nt insertions are indicated with lower case letters.

**Table 2:** Summary of rescue and tags expression of recombinant viruses harboring epitope tags.

Viral Protein	Site of Insertion (nt)	His	Flag	HA
CC	1902/1903	⚪	×	⚪
VPg-A	2016/2017	√	×	√
VPg-B	2033/2034	×	×	×
VPg-C	2199/2200	⚪	√	⚪
HVR	2261/2262	√	√	√

√ represents the recombinant virus that could be recovered and inserted into the tag of the recombinant virus that could be detected by IFA. ⚪ represents the recombinant virus that could be recovered and inserted into the tag of the recombinant virus that could not be detected by IFA. ^3^ × represents the recombinant virus that could not be recovered.

## Data Availability

The data used to support the findings of this study are available from the corresponding author upon request.

## Supplementary Materials

The following are available online at https://www.mdpi.com/article/10.3390/v13112119/s1, Table S1: The primers used in this study; Figure S1. Alignment of tag nucleotide sequences of P3, P6, P9, P12, and P15 recombinant viruses and parental virus (rMCMV-HDV).
