# Supplementary material for: Insertion of Exogenous Genes within the ORF1a Coding Region of Porcine Astrovirus
Source: Viruses. 2021 Oct 21;13(11):2119. doi: 10.3390/v13112119 (PMC8623754; doi:10.3390/v13112119)
Supplement: Supplementary file 1 [file viruses-13-02119-s001.zip › Supplementary Figure 1.pdf]

viruses

MDPI

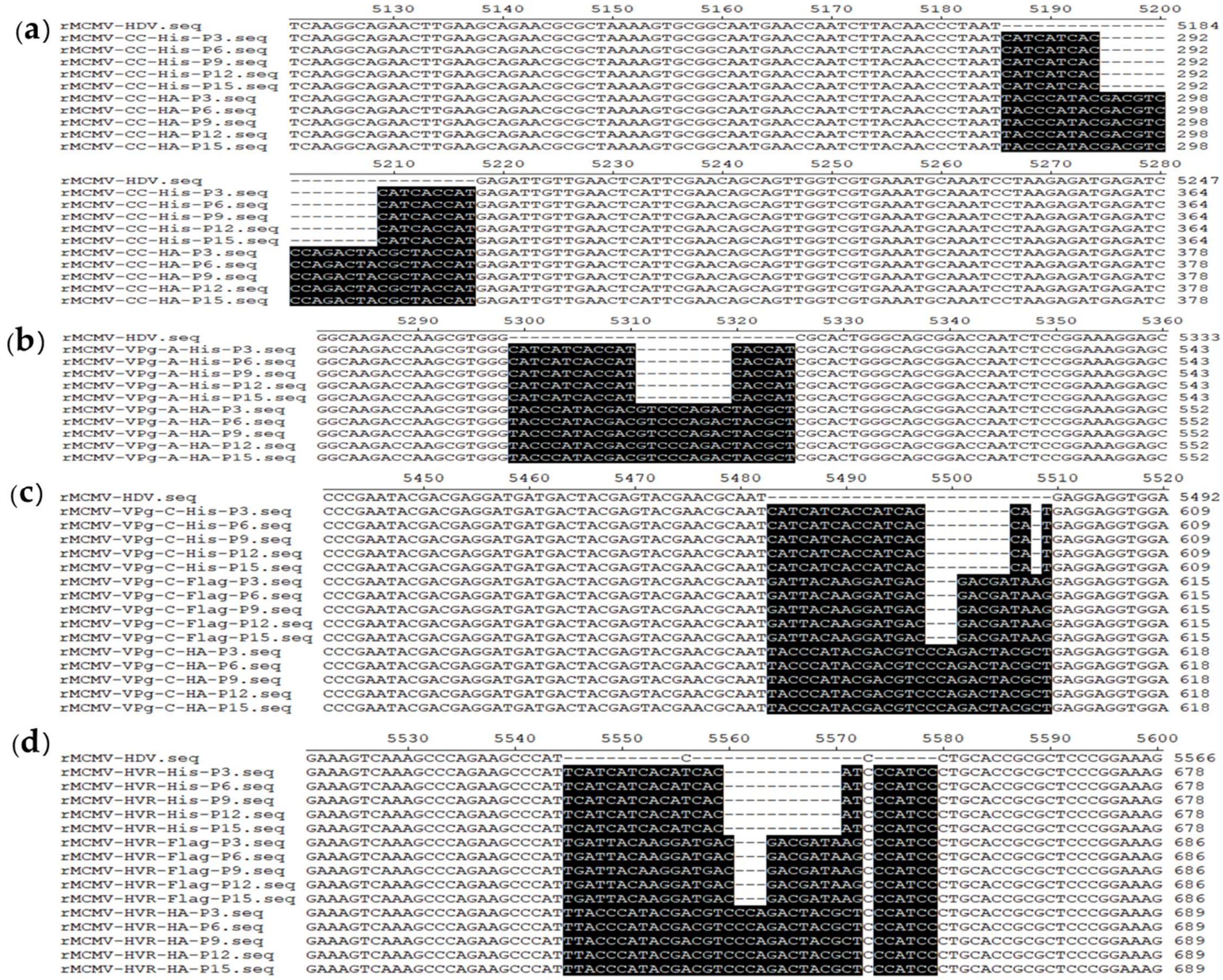

Supplementary Figure 1. Alignment of tag nucleotide sequences of P3, P6, P9, P12 and P15 recombinant viruses and parental virus (rMCMV-HDV). The shadows indicate the tag sequences.
