# Supplementary material for: Insertion of Exogenous Genes within the ORF1a Coding Region of Porcine Astrovirus
Source: Viruses. 2021 Oct 21;13(11):2119. doi: 10.3390/v13112119 (PMC8623754; doi:10.3390/v13112119)
Supplement: Supplementary file 1 [file viruses-13-02119-s001.zip › Supplementary Table 1.pdf]

Supplementary Table 1. The primers used in this study.

Primer name	Sequence(5'-3')	Use
CMVF	GCC*ACTAGT*TAGTTATTAATAGTAATCAATT	Replace the T7 promoter with the CMV promoter by overlap extension PCR
NgoR	AAGTTGAAGGCTGCATTGCGAGCA	
CMV23R	CCGGACTCTTTTGGAAGTTCACTAAACCAGCT	
Ngo23F	AGCTGGTTTAGTGAACTTCCAAAAGAGTCCGG	
F3F	AG*CTCGAG*GCATGCATTCTCA	Introduction of the ribozyme elements at the 3' end of the virus genome
P1	GGAGGTGGAGATGCCATGCCGACCCTTTTTTTTTTTTTTTTTTTTTTTTTTTTTTTTTTTTTTGCTTCTAATT	
P2	GAGTGGACGTGCGTCCTCCTTCGGATGCCCAGGTCGGACCGCGAGGAGGTGGAGATGCCATGCCGACCC	
P3	*CCGCGG*GCGCCCTCCCTTAGCCATCCGAGTGGACGTGCGTCCTCCTTC	
ORF1a-1606-F	TTG*TTAAC*GACGACATTGTGGTCTCAAC	Construction of transposon plasmid
ORF1a-2575-R	CT*AAGCTT*CTGAATCTTACTCCCAAG	
CC-His-F	CCAATCTTACAACCCTAATcatcatcaccatcaccatGAGATTGTTGAACTCATTC	Introduction of epitope tags in the CC domain
CC-His-R	GAATGAGTTCAACAATCTCatggtgatggtgatgatgATTAGGGTTGTAAGATTGG	
CC-Flag-F	CCAATCTTACAACCCTAATgattacaaggatgacgacgataagGAGATTGTTGAACTCATTC	
CC-Flag-R	GAATGAGTTCAACAATCTCcttatcgtcgtcatccttgtaatcATTAGGGTTGTAAGATTGG	
CC-HA-F	CCAATCTTACAACCCTAATtacccatacgacgtcccagactacgctGAGATTGTTGAACTCATTC	
CC-HA-R	GAATGAGTTCAACAATCTCagcgtagtctgggacgtcgtatgggtaATTAGGGTTGTAAGATTGG	
VPg-A-His-F	CAAGACCAAGCGTGGGcatcatcaccatcaccatCGCACTGGGCAGCGGACCAATC	Introduction of epitope tags in the VPg domain
VPg-A-His-R	GATTGGTCCGCTGCCCAGTGCGatggtgatggtgatgatgCCCACGCTTGGTCTTG	
VPg-A-Flag-F	CAAGACCAAGCGTGGGgattacaaggatgacgacgataagCGCACTGGGCAGCGGACC	
VPg-A-Flag-R	GGTCCGCTGCCCAGTGCGcttatcgtcgtcatccttgtaatcCCCACGCTTGGTCTTG	
VPg-A-HA-F	CAAGACCAAGCGTGGGtacccatacgacgtcccagactacgctCGCACTGGGCAGCGGACCAATC	
VPg-A-HA-R	GATTGGTCCGCTGCCCAGTGCGagcgtagtctgggacgtcgtatgggtaCCCACGCTTGGTCTTG	
VPg-B-His-F	GTGGGCGCACTGGGCAGCGGACTcatcatcaccatcaccatCGGACCAATCTCCGGAAAGGAGCTCG	
VPg-B-His-R	CGAGCTCCTTTCCGGAGATTGGTCCGatggtgatggtgatgatg	

	AGTCCGCTGCCCAGTGCGCCCAC	
VPg-B-Flag-F	GTGGGCGCACTGGGCAGCGGACTgattacaaggatgacgacgataagCGGACCAATCTCCGGAAAGGAGCTCG	
VPg-B-Flag-R	CGAGCTCCTTTCCGGAGATTGGTCCGcttatcgtcgtcatccttgtaatcAGTCCGCTGCCCAGTGCGCCCAC	
VPg-B-HA-F	GTGGGCGCACTGGGCAGCGGACTtacccatacgacgtcccagactacgctCGGACCAATCTCCGGAAAGGAGCTCG	
VPg-B-HA-R	CGAGCTCCTTTCCGGAGATTGGTCCGagcgtagtctgggacgtcgtatgggtaAGTCCGCTGCCCAGTGCGCCCAC	
VPg-C-His-F	CTACGAGTACGAACGCAATcatcatcaccatcaccatGAGGAGGTGGATTACGAC	
VPg-C-His-R	GTCGTAATCCACCTCCTCatggtgatggtgatgatgATTGCGTTCGTACTCGTAG	
VPg-C-Flag-F	CTACGAGTACGAACGCAATgattacaaggatgacgacgataagGAGGAGGTGGATTACGAC	
VPg-C-Flag-R	GTCGTAATCCACCTCCTCcttatcgtcgtcatccttgtaatcATTGCGTTCGTACTCGTAG	
VPg-C-HA-F	CTACGAGTACGAACGCAATtacccatacgacgtcccagactacgctGAGGAGGTGGATTACGAC	
VPg-C-HA-R	GTCGTAATCCACCTCCTCagcgtagtctgggacgtcgtatgggtaATTGCGTTCGTACTCGTAG	
HVR-His-F	GTCAAAGCCCAGAAGCCCATTcatcatcaccatcaccatCCCATCCCTGCACCGCGCTCCCGGAAAG	
HVR-His-R	CTTTCCGGGAGCGCGGTGCAGGGATGGGatggtgatggtgatgatgAATGGGCTTCTGGGCTTTGAC	
HVR-Flag-F	GTCAAAGCCCAGAAGCCCATTgattacaaggatgacgacgataagCCCATCCCTGCACCGCGCTCCCGGAAAG	Introduction of epitope tags in the HVR domain
HVR-Flag-R	CTTTCCGGGAGCGCGGTGCAGGGATGGGcttatcgtcgtcatccttgtaatcAATGGGCTTCTGGGCTTTGAC	
HVR-HA-F	GTCAAAGCCCAGAAGCCCATTtacccatacgacgtcccagactacgctCCCATCCCTGCACCGCGCTCCCGGAAAG	
HVR-HA-R	CTTTCCGGGAGCGCGGTGCAGGGATGGGagcgtagtctgggacgtcgtatgggtaAATGGGCTTCTGGGCTTTGAC	
HVR-AflII-F	GTCAAAGCCCAGAAGCCCATTC*TTAAG*GCC*GTCGAC*CCCATCCCTGCACCGCGCTCCCGGAAAG	Introduction of restriction sites in the HVR domain
HVR-SalI-R	CTTTCCGGGAGCGCGGTGCAGGGATGGG*GTCGAC*GGCC*TTAAG*AATGGGCTTCTGGGCTTTGAC	
iLOV-AflII-F	GC*CTTAAG*ATGATAGAGAAGAATTTCGTC	Amplification of iLOV sequence
iLOV-SalI-R	GC*GTCGAC*TACATGATCACTTCCATCGAGC	

[1] Epitope tags are shown as lower case letters.

[2] Restriction sites are shown as italics.
